# Intestinal strongyloidiasis: radiological findings that support the diagnosis

**DOI:** 10.1590/0100-3984.2015.0175

**Published:** 2017

**Authors:** José Henrique Frota Júnior, Marcos Antônio Haddad Pereira, Paulo Gustavo Maciel Lopes, Leandro Accardo Matos, Giuseppe D'Ippolito

**Affiliations:** 1 Escola Paulista de Medicina da Universidade Federal de São Paulo (EPM-Unifesp), São Paulo, SP, Brazil

**Figure 1:**
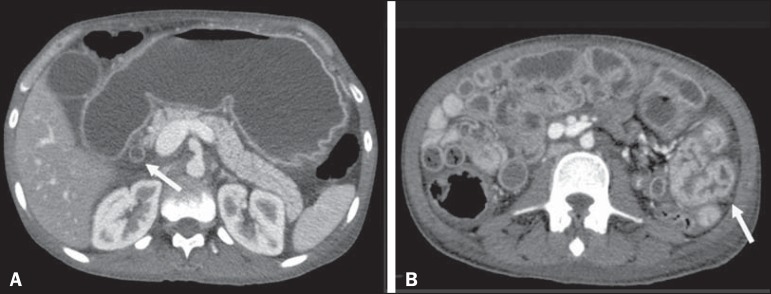
Intravenous contrast-enhanced CT scan showing accentuated gastric distension with mucous enhancement, dilation of the bile duct (arrow in **A**) and thickening of the intestinal wall in segments of the small intestine (arrow in **B**), with fluid distension of the intestinal loops.

**Figure 2:**
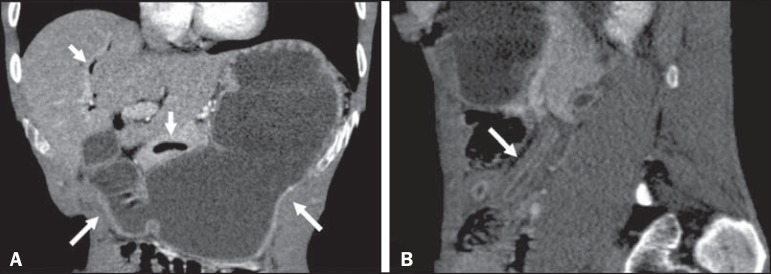
Intravenous contrast-enhanced CT scan, in the coronal and sagittal planes (**A** and **B**, respectively), showing accentuated gastric and duodenal distension (long arrows in **A**), accompanied by gas in the biliary tract and pancreas (short arrows in **A**), together with the "lead pipe" sign, characterized by thickening of the walls, rigidity, and luminal narrowing of the small intestine (arrow in **B**).

Dear Editor,

Two male patients, 38 and 32 years of age (patients 1 and 2, respectively), sought treatment with complaints and the clinical/biochemical profile described below. *Patient 1* – This patient complained of nausea and intermittent postprandial vomiting, for approximately two months, accompanied by mild abdominal pain, diarrhea, and weight loss. Physical examination revealed emaciation, with discrete edema of the lower limbs. Laboratory tests showed a low albumin level (0.9 g/dL) and an elevated level of C-reactive protein (31.4 mg/L). A computed tomography (CT) scan of the abdomen showed diffuse thickening of the intestinal wall in segments of the small intestine, more accentuated in the region of the jejunum and the second portion of the duodenum, together with gastric distension, thickening (with enhancement) of the mucous membrane, dilation of the bile duct (Figure 1), and free fluid in the peritoneal cavity. *Patient 2* – This patient also complained of nausea and postprandial vomiting, accompanied by mild abdominal pain, for one month, exacerbated for one day. Physical examination revealed emaciation, with dull pain on abdominal palpation. Laboratory tests showed discrete leukocytosis without deviation, a low albumin level (2.2 g/dL), and an elevated level of C-reactive protein (65.1 mg/L). A CT scan showed accentuated thickening of the intestinal wall in segments of the jejunum, together with upstream gastric and duodenal dilation, discrete dilation of the bile duct, pneumobilia, and gaseous foci in the main pancreatic duct (Figure 2). In both of the cases presented here, the diagnosis of strongyloidiasis was confirmed by gastroduodenal biopsy and by parasitological examination of the feces. Both patients were treated with support measures and ivermectin, which resulted in significant improvement of their symptoms.

*Strongyloides stercoralis* is an intestinal helminth endemic to many regions with tropical or subtropical climates, as well as some temperate regions^(1-3)^. Infection with *S. stercoralis* (strongyloidiasis) can manifest as a clinical syndrome involving the skin, lungs, or gastrointestinal tract^(3)^. Approximately half of all cases of *S. stercoralis* infection are asymptomatic^(4,5)^. When present, the symptoms of strongyloidiasis are vague and can include abdominal pain, diarrhea, nausea, and vomiting^(1)^. Less frequently, the disease can manifest as malabsorption syndromes, paralytic ileus, intestinal obstruction (possibly related to pneumobilia), or gastrointestinal bleeding^(4-6)^.

In *S. stercoralis* infection, the imaging findings of the alterations to the large intestine are nonspecific and similar to those seen in inflammatory/infectious intestinal diseases of other causes, especially the edema of the duodenal wall and of the proximal small intestine, as well as mucous congestion, coarse folds, and dilation of the bowel loops^(7-9)^. At that stage, the radiological images are similar to those seen in hypoalbuminemia, ascites and peritonitis. The combination of dilation of the stomach and thickening of the gastric mucous membrane is less common in inflammatory processes of other causes and, in strongyloidiasis, results in luminal narrowing and thickening of the duodenal folds, producing a "lead pipe" sign (Figure 2B), and upstream distension, as seen in the cases presented here^(9)^. In some cases, there can be reflux of oral contrast into the biliary tree or pneumobilia, due to sphincter of Oddi dysfunction, caused by severe inflammation of the duodenal wall, as was observed in our patient 2^(10)^. To our knowledge, there have been no reported cases of gas in the main pancreatic duct, although the cause should be the same as that of pneumobilia.

When there are intestinal manifestations, the main differential diagnoses of strongyloidiasis include Crohn's disease, lymphoma, tuberculosis, and other causes of enterocolitis. Laboratory tests and some tomographic images, as well as the extensive lymphadenopathy seen in lymphoma and the necrotic lymph nodes seen in tuberculosis, can facilitate the distinction among the diseases^(11)^. Because strongyloidiasis presents a nonspecific clinical profile, it can evolve to a disseminated form, with sepsis and shock, especially in immunosuppressed patients^(12)^. The diagnosis of strongyloidiasis should be suspected and confirmed early on, through the analysis of some of the radiological signs described here. The definitive diagnosis is based on a finding of larvae in the feces, tracheal sections, bronchial lavage fluid, gastric aspirate, or biopsy samples-from the stomach, jejunum, skin, or lung^(12)^. Intestinal strongyloidiasis is an important differential diagnosis of inflammatory diseases of the small intestine and should be considered in the presence of certain clinical aspects and a combination of imaging findings, including thickening/enhancement of the mucous membrane in the small intestine, gastric distension, and biliopancreatic changes such as dilation and gas within the biliary tract and pancreas.
